# Association between albumin infusion and septic patients with coronary heart disease: A retrospective study based on medical information mart for intensive care III database

**DOI:** 10.3389/fcvm.2022.982969

**Published:** 2022-10-19

**Authors:** Zhiwen Ye, Ming Gao, Chenglong Ge, Wenrui Lin, Lina Zhang, Yu Zou, Qianyi Peng

**Affiliations:** ^1^Department of Critical Care Medicine, Xiangya Hospital, Central South University, Changsha, China; ^2^Hunan Provincial Clinical Research Center for Critical Care Medicine, Xiangya Hospital, Central South University, Changsha, China; ^3^National Clinical Research Center for Geriatric Disorders, Xiangya Hospital, Central South University, Changsha, China; ^4^Department of Geriatric Medicine, Center of Coronary Circulation, Xiangya Hospital, Central South University, Changsha, China; ^5^Department of Dermatology, Xiangya Hospital, Central South University, Changsha, China; ^6^Department of Anesthesiology, Xiangya Hospital, Central South University, Changsha, China

**Keywords:** coronary heart disease, sepsis, albumin infusion, resuscitation, mortality

## Abstract

Coronary heart disease (CHD) is a common comorbidity in intensive care unit (ICU) patients, particularly in the elderly. This particular population may have worse conditions during sepsis, and it presents an overwhelming challenge for clinical practice. Previous studies suggested that patients with CHD have an increased risk of cardiovascular events, and low albumin concentration worsens the prognosis of patients with stable CHD. Hypoalbuminemia in patients with sepsis is common due to nutritional disorders, excessive consumption, and leakage. Albumin is a fluid often used for resuscitation in patients with sepsis. However, albumin infusion in patients with sepsis and CHD has rarely been studied. The effects and safety of albumin infusion in patients with sepsis and CHD remain unclear. Therefore, we collected medical information from Mimic-III (Mimic-III) and compared the all-cause mortality and cardiovascular mortality at 28- or 90-day between the albumin and non-albumin groups in septic patients with CHD. A total of 2,027 patients with sepsis and CHD were included in our study, with 405 in the albumin group and 1,622 in the non-albumin group. After propensity score matching (PSM), 350 pairs were included in our study. Improved survival benefits were found in the albumin group at the 28-day all-cause mortality compared with the non-albumin group (hazard ratio [*HR*], 0.54; 95% *CI*: 0.38–0.78; *p* = 0.0009). However, no difference was detected in the 90-day survival benefits (*HR*, 0.80, 95% *CI*: 0.60–1.06, *p* = 0.1207). Albumin infusion did not reverse cardiovascular mortality neither at 28th day nor at 90th day (cardiovascular mortality: 28 days, *HR*, 0.52, 95% *CI*: 0.23–1.19, *p* = 0.1218; 90 days, *HR*, 0.66, 95% *CI*: 0.33–1.33, *p* = 0.2420).

## Introduction

Sepsis is defined as an abnormal inflammatory response to infection that leads to life-threatening organ dysfunction in the host (1). It is a common but challenging disorder for intensive care unit (ICU) doctors. Patients who develop sepsis have poor outcomes and are the leading cause of mortality in the ICU (2). Chronic conditions may increase mortality in patients with sepsis (3). Coronary heart disease (CHD) is a common comorbidity in patients with sepsis and results in fatal incidents (4). A systemic review suggested that patients with CHD were at a high risk of cardiovascular incidences if aggravated by sepsis (5). Fluid administration is an important intervention in sepsis resuscitation. Colloids are often chosen as they remain in the intravascular space for a longer time than crystalloids, especially in the condition of increased endothelial permeability in sepsis. Albumin is the preferred colloid in sepsis, as fluid resuscitation with albumin is less likely to cause nephrotoxicity than with artificial colloids (6). Albumin administration is often used in two conditions in sepsis. First, it is often used in the initial resuscitation stage. Albumin infusion at this stage increases plasma colloid osmotic pressure and its volume expanding capacity helps in maintaining hemodynamic stability (7). In addition, it has many physiological functions, such as antioxidant, anti-inflammatory, anticoagulant, and antiplatelet aggregation effects, protecting glycocalyx, facilitating microcirculation, and acid-base balance (6, 8). The Surviving Sepsis Campaign Guidelines recommended that septic patients with substantial crystalloid input used albumin during initial resuscitation (9). Second, albumin is in the fluid removal stage to help in achieving the negative fluid balance. The production and metabolism of serum albumin are severely disrupted in patients with sepsis. Massive resuscitation leads to fluid retention, and edema formation is worsened by hypoalbuminemia. It was found that the administration of albumin increased the effect of furosemide due to changes in renal hemodynamics (10). In patients with acute lung injury, a combination of a diuretic with albumin had a better effect than furosemide alone (11).

Regarding the effect of albumin on the prognosis of patients with sepsis, the Saline versus Albumin Fluid Evaluation (SAFE) study determined that albumin infusion did not damage renal or other organs and may reduce the risk of mortality (12). However, a multicenter randomized controlled trial suggested that albumin infusion as resuscitation for patients with sepsis (with or without baseline hypoalbuminemia) failed to provide survival benefits (13). Furthermore, albumin administration to critically ill patients may lead to cardiovascular incidents and even excess mortality (14). The effect of albumin on the occurrence of cardiac complications is seldom described in patients with sepsis. Epidemiological evidence showed that low serum albumin levels were linked to incident ischemic heart disease, heart failure, atrial fibrillation, stroke, and venous thromboembolism, independent of risk factors, body mass index, and inflammation. Hypoalbuminemia has emerged as an independent prognosticator in many cardiovascular diseases, such as coronary artery disease, heart failure, congenital heart disease, infective endocarditis, and stroke, even after adjusting for usual causal factors and prognostic markers (15). Therefore, albumin administration is often used in cardiovascular disease patients with hypoalbuminemia and edema, however, it also increases the load of the heart and may cause congestive heart failure and other cardiac complications. It was reported that, in patients with congestive heart failure, the albumin infusion group had markedly higher in-hospital mortality (36.42% vs. 21.81%), longer ICU length of stay (LOS), and hospital LOS. Albumin infusion was significantly associated with an increased risk of in-hospital mortality (16). In patients with subarachnoid hemorrhage, the maximum tolerated dosage of albumin was determined by the rate of treatment-related severe or life-threatening heart failure. Dosages higher than 1.25 g/kg/day were associated with significant cardiovascular complications (17). In patients with acute ischemic stroke, adverse events related to albumin infusion were those related to volume overload leading to acute heart failure or pulmonary edema (18).

Thus, the effects and safety of albumin infusion in patients with sepsis, especially those with the basic cardiovascular disease, remain controversial (19). Inconsistent with previous studies, we conducted a study specific to septic patients with CHD to further clarify the link between albumin input and outcomes in septic patients with CHD. We retrospectively collected data on septic patients with CHD from the Medical Information Mart for Intensive Care III (MIMIC-III) database and compared mortality and cardiovascular mortality at 28 and 90 days with or without albumin treatment. We used PSM to guarantee that our cohorts were comparable so that we could draw a more reliable conclusion. The Kaplan–Meier survival analyses were used to compare end events. We evaluated confounding factors and avoided potential bias through a multivariate analysis using Cox regression. Finally, a subgroup analysis was performed to assess the heterogeneity between subgroups.

## Materials and methods

### Study design

This was a single-center retrospective study. Information was collected from the database based on the Medical Information Mart for Intensive Care III (MIMIC-III) (20). MIMIC-III is an open-access database. It recorded data related to patients in the ICU at the Beth Israel Women’s Dickens Medical Center from 2001 to 2012 and contained medical health data for more than 40,000 patients. Our study was designed to investigate whether albumin application could improve the mortality of patients with sepsis and CHD. Our project was authorized by the Massachusetts Institute of Technology (MIT), and we obtained a certificate from the Beth Israel Deaconess Medical Center (BIDMC). A total of 46,476 patients with first-time ICU admission from the MIMIC-III database were eligible for inclusion. A total of 10,179 patients met the definition of Sepsis 3.0 criteria. Patients who presented with infection and with a total score ≥ 2 points on the Sequential Organ Failure Assessment were identified as having sepsis. CHD conforms to the International Classification of Diseases 9th Edition (ICD-9) code in MIMIC-III. Patients aged less than 18 years, with ICU stays of not more than 24 h, or those with a high risk for albumin use, such as systolic heart failure or pulmonary edema, were excluded. The filtering process is described in Supplementary Figure 1. To avoid potential bias and balance the baseline population, we used the propensity score matching (PSM) method with a 1:1 proportion to screen our targets. Finally, 700 patients were enrolled in our study and divided into the albumin infusion group and the non-albumin infusion group based on whether they received albumin therapy after admission.

### Data extraction

The following data were extracted from the MIMIC-III database using the Structured Query Language (SQL): age, sex, weight, and basic information; severity of illness included Glasgow Coma Score (GCS), Sequential Organ Failure Assessment (SOFA) score, and Simplified Acute Physiology Score II (SASPII) within the first 24 h of ICU admission; interventions within 24 h of ICU admission; ventilation use, vasopressor use, renal replacement therapy (RRT) use, comorbidities, hypertension, diabetes, and chronic obstructive pulmonary disease (COPD); laboratory tests, white blood cell (WBC) count, platelet, glucose, creatinine, blood urea nitrogen (BUN), international normalized ratio (INR), prothrombin time (PT), baseline serum albumin, type of sepsis infection, affected organs, and fluid infusion in the first 24 h following ICU admission. Vital signs, lactate, and SOFA scores before death or discharge are shown in Supplementary Table 1.

### Primary outcomes and secondary outcomes

The primary outcomes (POs) were 28- and 90-day all-cause mortality from admission. All death information was obtained from hospitalization and follow-up data. The secondary outcomes (SOs) were 28- and 90-day cardiovascular mortality. Demise in patients with a major diagnosis of cardiovascular incidents was defined as cardiovascular mortality, such as myocardial infarction, cerebral infarction, and cerebral hemorrhage. All the diagnoses in our study originated from MIMIC-III and also accorded with the category of ICD-9 codes.

### Statistical analysis

Septic patients with underlying CHD were divided into two groups: the albumin infusion group and the non-albumin infusion group. We adjusted for confounding and potential selection bias between the two groups using the propensity score and the 1:1 nearest neighbor matching method (21). Comparisons of baseline characteristics are listed in Table 1. We performed independent sample Student’s tests on the continuous variables conforming to a Gaussian distribution and the Mann–Whitney *U*-test for those not belonging to a Gaussian distribution. The chi-square analysis was used to analyze categorical variables.

**TABLE 1:** Comparisons of baseline characteristics for patients with albumin infusion or not.

Characteristics	Albumin infusion		*P*-value
	Yes	No	
*N*	350	350	
Age, mean (SD)	74.02 (10.43)	74.45 (11.66)	0.607
Women (%)	149 (42.6)	146 (41.7)	0.878
Weight (kg)	82.61 (21.00)	82.40 (25.07)	0.904
**Severity of illness**			
GCS score	13.47 (3.37)	13.35 (3.13)	0.634
SOFA score	6.25 (3.41)	6.35 (3.42)	0.699
SASPII score	44.90 (14.13)	45.35 (14.84)	0.682
**Interventions, *n* (%)**			
Ventilation use (1st 24 h)	244 (69.7)	236 (67.4)	0.569
Vasopressors use (1st 24 h)	274 (78.3)	265 (75.7)	0.472
RRT use (1st 24 h)	18 (5.1)	25 (7.1)	0.345
**Comorbidities, *n* (%)**			
Hypertension	170 (48.6)	187 (53.4)	0.226
Diabetes	156 (44.6)	167 (47.7)	0.448
COPD	14 (4.0)	17 (4.9)	0.713
**Laboratory tests, mean (SD)**			
WBC (× 109/L)	16.49 (7.52)	16.64 (9.42)	0.815
Platelet (×109/L)	238.07 (120.90)	232.84 (108.75)	0.547
Glucose (mg/dL)	191.98 (78.27)	191.57 (80.84)	0.946
Creatinine (mg/dL)	1.65 (1.30)	1.71 (1.39)	0.558
BUN (mg/dL)	32.07 (20.28)	32.92 (21.67)	0.592
INR (ratio)	1.70 (1.70)	1.81 (1.68)	0.375
PT (s)	17.60 (6.59)	18.36 (12.61)	0.322
Serum albumin (g/L)	2.94 (0.57)	2.99 (0.46)	0.245

GCS, Glasgow Coma Scale; SOFA, Sequential Organ Failure Assessment; SAPSII, Simplified Acute Physiology Score II; RRT, Renal Replacement Therapy; COPD, Chronic Obstructive Pulmonary Disease; WBC, White Blood Cell; BUN, Blood Urea Nitrogen; INR, International Normalized Ratio; PT, Prothrombin Time; Baseline serum albumin.

The cumulative survival rates at 28 and 90 days for total mortality or cardiovascular mortality were calculated using the Kaplan–Meier survival analyses and the log-rank test. The Cox proportional regression analysis was used to evaluate the independent correlation between albumin infusion and outcomes, shown as hazard ratios (*HR*s) with 95% confidence intervals (*CI*s). Model 1 (the original analysis) was not adjusted. Considering age and sex, we obtained an adjusted analysis using Model 2. Based on Model 2, we calculated the adjusted *HR* and 95% *CI* by adding weight, GCS score, SOFA score, SASPII score, ventilation use (1st 24 h), vasopressor use (1st 24 h), RRT use (1st 24 h), hypertension, diabetes, COPD, WBC, platelets, glucose, creatinine, BUN, INR, infection of sepsis, affected organ, and fluid infusion at the first 24 h. The values of *p* of both tails less than 0.05 were regarded as statistically significant.

To further investigate the effect of improving 28-day mortality between the albumin infusion and non-albumin infusion groups, we performed a subgroup analysis for age, sex, hypertension, and diabetes. We assessed potential confounders using a multivariate analysis with COX regression among the subgroups. The interaction between subgroup variables and interventions was included in our study. All data cleaning and management were conducted using Stata 15.0. Data analysis and diagramming were performed using R 4.1.1.

## Results

### Baseline characteristics of patients

Comparisons of baseline characteristics between the albumin infusion and non-albumin infusion groups are listed in Table 1. In total, 350 patients were enrolled in each group. The variables with potential selection bias and significant misunderstanding results were well matched by the propensity score and the 1:1 nearest neighbor matching method, including the following factors within the first 24 h of ICU admission: age, sex, weight, GCS score, SOFA score, and SASPII, interventions, comorbidities, the maximum value of laboratory tests (WBC, platelet, glucose, creatinine, BUN, INR, and PT), and the baseline serum albumin (Table 1). The total dose of albumin infusion in the albumin group was 25.19 g during their hospitalization. During the first day of resuscitation, the fluid intake was significantly more in the albumin group than in the non-albumin group (4,674.08 vs. 3,689.70 ml, *p* < 0.001). In addition, the proportion of abdominal infection in the non-albumin input group was higher than that in the albumin group (*n* = 96 vs. 58, *p* < 0.001) (Supplementary Table 2). We adjusted the source of infection type and fluid intake in the multivariate regression model to test our results.

### Effects of albumin infusion on the primary and secondary outcome

**FIGURE 1:**
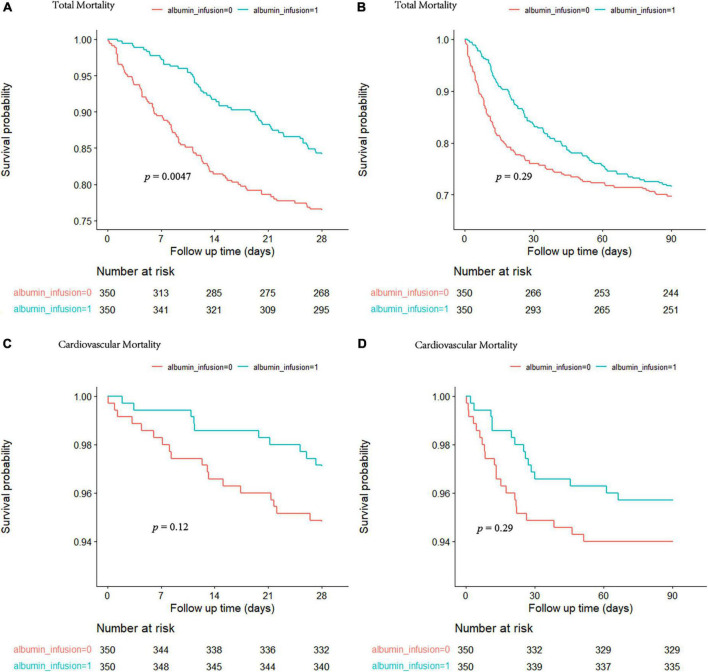
The cumulative survival rate at 28 and 90 days for total mortality **(A,B)** and cardiovascular mortality **(C,D)** was calculated by the Kaplan-Meier survival analyses and the log-rank tests. The red curve represents the non-albumin infusion group. The green curve presents the albumin infusion group.

The Kaplan–Meier analysis showed a higher survival probability at 28 days of total mortality in patients who received albumin infusion treatment during their admission (Figure 1A). In addition, there was a statistically significant difference in 28-day mortality between the albumin infusion and non-albumin infusion groups (*p* = 0.0047). However, no significant difference was detected between the two groups in the 90-day survival probability (Figure 1B, *p* = 0.29). To further assess the effects of albumin infusion on septic patients with CHD, we compared the 28- and 90-day mortality for cardiovascular incidents (Figures 1C,D). Neither 28- nor 90-day cardiovascular mortality showed a significant difference between the two groups (*p* = 0.12 and *p* = 0.29, respectively).

**FIGURE 2:**
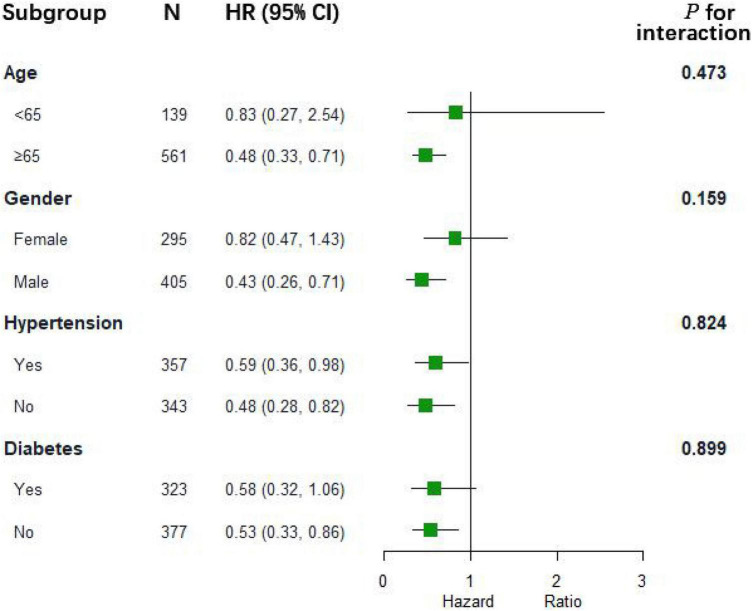
The association between albumin infusion and 28-day mortality in subgroups. HR hazard ratio; and CI confidence interval.

As shown in Table 2, we further assessed the prognostic risk between the two groups using the Cox proportional hazards model. We obtained a consistent conclusion with the Kaplan–Meier analysis in Models 1, 2, and 3 that patients in the albumin group had a better 28-day survival probability than those in the non-albumin group (Model 3: *HR*, 0.54; 95% *CI*: 0.38–0.78; *p* = 0.0009). However, with regard to the 90-day total mortality and cardiovascular mortality at 28 or 90 days, albumin infusion had no improved outcome compared with the non-albumin group (Model 3: total mortality at 28 days, *HR*, 0.80, 95% *CI*: 0.60–1.06, *p* = 0.1207; cardiovascular mortality: 28 days, *HR*, 0.52, 95% *CI*: 0.23–1.19, *p* = 0.1218; 90 days, *HR*, 0.66, 95% *CI*: 0.33–1.33, *p* = 0.2420). The subgroup analysis evaluated the heterogeneity of albumin therapy in patients based on age, sex, hypertension, and diabetes (Figure 2). We could not find evidence to support the hypothesis that albumin infusion is more profitable among these subgroups.

**TABLE 2:** Risk of primary outcomes (POs) and secondary outcomes (SOs) for patients with albumin infusion or not.

		Model 1		Model 2		Model 3	
		HR (95% CI)	*P-*value	HR (95% CI)	*P-*value	HR (95% CI)	*P-*value
Total mortality	28 day	0.61 (0.46, 0.86)	0.005	0.62 (0.44, 0.87)	0.006	0.54 (0.38, 0.78)	0.0009
	90 day	0.86 (0.66, 1.13)	0.290	0.87 (0.66, 1.14)	0.314	0.80 (0.60, 1.06)	0.1207
Cardiovascular mortality	28 day	0.55 (0.25, 1.18)	0.126	0.54 (0.25, 1.18)	0.123	0.52 (0.23, 1.19)	0.1218
	90 day	0.70 (0.36, 1.36)	0.295	0.70 (0.36, 1.36)	0.291	0.66 (0.33, 1.33)	0.2420

Model 1: unadjusted. Model 2: adjusted for baseline age and gender. Model 3: add Weight, GCS score, SOFA score, SASPII score, ventilation use (1st 24 h), vasopressors use (1st 24 h), RRT use (1st 24 h), hypertension, diabetes, COPD, WBC, platelet, glucose, creatinine, BUN, INR, PT, baseline serum albumin, type of sepsis infection, affected organs, and fluid infusion in the first 24 h on admission to the intensive care unit (ICU) to Model 2. HR, hazard ratio; and CI, confidence interval.

## Discussion

Albumin, a natural colloid, is the most abundant small-molecule protein in the blood. Colloids are macromolecules for which the vessel wall has a low permeability. Therefore, less volume is required for an equal plasma volume expansion compared with crystalloids (22). The Surviving Sepsis Campaign Guidelines recommended that septic patients with substantial crystalloid input used albumin to maintain stable arterial pressure during initial resuscitation (9). However, adverse events related to albumin infusion were those related to volume overload leading to acute heart failure or pulmonary edema. Dosages higher than 1.25 g/kg/day were associated with significant cardiovascular complications (17). Similar results were observed in Ginsberg’s study (18). Thus, the effects and safety of albumin infusion in patients with sepsis, especially those with a basic cardiovascular disease, remain controversial.

Increasing evidence suggests that the effect of albumin treatment may be heterogeneous in patients with sepsis, particularly in those with organic heart disease. The common clinical characteristics of sepsis, such as hemodynamic instability and generalized microcirculation disorders, can lead to myocardial ischemia and cardiovascular depression, especially in patients with CHD (23). The National Institutes of Health (NIH) conference indicated a significantly increased mortality rate of 70–90% compared with 20% in septic patients without cardiovascular impairment (24). These data support that patients with sepsis and CHD had a worse life-threatening risk and needed to be treated individually. Previous studies have not reached a consensus on whether albumin infusion could improve survival benefits for critically ill patients. Furthermore, evidence of the association between albumin infusion and cardiovascular mortality is rare.

Although a study by Rochwerg et al. (25) found that albumin infusion reduced mortality during resuscitation in patients with septic shock. However, septic patients with CHD were not included, and the effect and safety of albumin in this population remain unclear. Therefore, we retrospectively compared the prognosis of albumin infusion in septic patients with CHD at 28 and 90 days and cardiovascular mortalities.

Our results showed that survival benefits were found in the albumin group at the 28-day all-cause mortality compared with the non-albumin group (*HR*, 0.54; 95% *CI*: 0.38–0.78; *p* = 0.0009). However, no difference was detected in the 90-day survival benefits (*HR*, 0.80, 95% *CI*: 0.60–1.06, *p* = 0.1207). This further provides evidence and safety for those with potential benefits from albumin input.

According to the Sepsis 3.0 diagnostic guideline, infection and organ dysfunction are the most critical features of sepsis. For this reason, we selected essential metrics for the outcome on the patient with septsis. The severity of systemic illness was assessed with the use of the Simplified Acute Physiology Score, with scores ranging from 0 to 163 and higher scores indicating more severe illness. It covers a multi-dimensional comprehensive evaluation of blood pressure, respiration, urine volume, serum potassium, serum sodium, blood bicarbonate, and inflammatory cells. Organ function was assessed daily with the use of the Sequential Organ Failure Assessment (SOFA) score, which ranges from 0 to 4 for each of the five components (respiratory, coagulation, liver, cardiovascular, and renal components), with higher scores indicating more severe organ dysfunction. As shown in Table 1, all these figures are well matched after PSM. Furthermore, we added a Cox proportional hazard model to the study, which made our results more reliable and avoided potential confounding bias as much as possible. There was no difference detected in the baseline serum albumin between the two groups (albumin infusion group vs. non-albumin infusion group, 2.94 vs. 2.99 g/L, *p* = 0.245, Table 1). The mean albumin dose infusion to the patient was 25.19 g during their hospitalization. During the first day of resuscitation, our study showed that fluid intake was higher in the albumin group than in the non-albumin group (4,674.08 vs. 3,689.70 ml, *p* < 0.001, Supplementary Table 2). It is possible that albumin solution has a certain capacity of its own, resulting in higher fluid infusion in the albumin group.

Similarly, we found that the proportion of abdominal infection in the albumin input group was higher than that in the non-albumin group (*n* = 96 vs. 58, *p* < 0.001, Supplementary Table 2). To control for the effect of confounders on outcomes, we adjusted for the source of infection type and fluid intake in the multivariate regression model. Our results suggested an improved survival benefit at 28-day mortality in septic patients with CHD between the albumin infusion group and the non-albumin infusion group (*HR*, 0.54; 95% *CI*: 0.38–0.78; *p* = 0.0009).

Critical information before death or discharge, such as body temperature, heart rate, mean arterial pressure, lactate concentration, and the SOFA score, was supplemented, as shown in Supplementary Table 1. No statistical difference was detected in vital signs between the albumin and non-albumin groups, similar to previous studies (18). The possible reason is that, after initial adequate fluid resuscitation, and effective anti-infective treatment, the patient’s vital signs recovered relatively smoothly. In addition, patients who died accounted for only a small part in our study cohort, and it may be that the changes in these patients did not change the target level of the entire cohort. There was no significant difference in the SOFA score between the two groups after resuscitation with or without albumin fluid (5.03 vs. 4.83, *p* = 0.468, Supplementary Table 1). Fluid resuscitation of albumin is critical in the early stages (26), especially in patients who require abundant crystalloid resuscitations, but given that patients with early sepsis are always in a state of multiple organ failure, early albumin infusion effects are difficult to detect. Therefore, we suggest that in follow-up studies on albumin during resuscitation, the focus of efficacy observation should be on the early stage.

Myocardial ischemia is aggravated in patients with CHD due to sepsis, which may cause cardiac depression and increase the mortality of patients with sepsis. Systemic inflammation leads to the dysfunction of endothelial cells (27) and abnormalities in vascular permeability (28) that worsens microcirculation disorders. These alterations may accelerate cardiac ischemia and cause a deterioration of the condition. Due to its anti-inflammatory, antioxidant, anti-apoptotic, anticoagulant, and antiplatelet aggregation properties (29), albumin infusion protects endothelial function (30), restores vascular permeability, and improves the microvasculature (28, 31). In addition, albumin infusion participates in fluid balance because of its powerful function in preventing fluid in vascular flow into the third interstitial space. This ensures fluid volume stability (32), which provides sufficient infusion for coronary arteries, especially in systemic hypotension conditions, and significantly reduces systemic vascular resistance typically encountered in sepsis (33). Epidemiological evidence showed that hypoalbuminemia was an independent prognosticator in cardiovascular complications (4). These mechanisms may explain our findings of an improvement in the 28-day outcome in septic patients with CHD. However, inconsistent with the CRISTAL randomized trial, which showed a lower mortality rate after albumin therapy for patients with sepsis at 90-day mortality (34), our study found no difference between the two groups regarding 90-day mortality survival benefits in septic patients with CHD. We used the SOFA score and SAPSII in the first 24 h to evaluate physiological parameters and health status. However, as the length of hospital stay increased, mortality was attributed more to chronic status and emerging complications instead of acute physiological alterations, leading to a weaker curative effect. Robust systemic inflammation response and vascular resistance are mainly focused on the early stage of sepsis, which may result in global ischemia, increasing the risk of mortality in septic patients with CHD (5). Patients with a longer ICU stay were related to malnutrition and reduced baseline albumin levels, which decreased the therapeutic effect. All these factors may help to understand the difference in mortality between 28 and 90 days.

Cardiovascular mortality was defined as a major cause of mortality, such as myocardial infarction, stroke, and encephalorrhagia. Albumin administration did not reverse cardiovascular mortality. According to our study, there was no difference in cardiovascular mortality between the albumin and non-albumin groups at 28 or 90-day cardiovascular mortality. A potential explanation for this phenomenon is that albumin infusion prevented the development of cardiovascular incidents in septic patients with CHD and thus reduced mortality but did not reverse the mortality relative to cardiovascular events once this process occurred. Our study had no direct evidence to demonstrate that albumin reduced the risk of cardiovascular incidents in septic patients with CHD through anti-inflammation, anti-apoptosis, and protection of endothelial cells, thus contributing to a lower mortality rate in the albumin therapy group. Our research demonstrated an improved 28-day survival rate associated with albumin infusion in this specific population. More randomized controlled trials are required to further support this finding.

## Limitations

Our study has some limitations. First, it was a retrospective study, and no definitive causal relationship could be established. Second, our research is based on an open-access database, and some missing values exist. However, we balanced our baseline characteristics with a propensity score and a 1:1 nearest-neighbor matching method to match our groups well and reduce possible selective bias. Finally, we did not collect the adverse events resulting from albumin infusion, but our study excluded the specific population that had a possible adverse event for albumin use, such as systolic heart failure and severe pulmonary edema.

## Conclusion

The 28-day all-cause mortality improved after albumin administration in patients with sepsis and CHD. However, no survival benefits were observed in the albumin therapy group for 90-day mortality. In addition, our research showed that albumin fusion did not reverse the adverse events neither at 28-day nor at 90-day cardiovascular mortality. Our research was a retrospective study and provided only preliminary data for future investigations. Further randomized clinical trials and clinical practice are needed to validate our findings.

## Data availability statement

The original contributions presented in this study are included in the article/Supplementary material, further inquiries can be directed to the corresponding author/s.

## Ethics statement

This study was an analysis of a third-party anonymized publicly available database with pre-existing institutional review board (IRB) approval. The Institutional Review Boards at the Beth Israel Deaconess Medical Center (protocol 2001-P-001699/14) and Massachusetts Institute of Technology (protocol 0403000206) have approved the data collection and the use of MIMIC-III for research purposes and granted waiver of informed consent. Written informed consent for participation was not required for this study in accordance with the national legislation and the institutional requirements. Written informed consent was not obtained from the individual(s) for the publication of any potentially identifiable images or data included in this article.

## Author contributions

ZY finished the original manuscript. MG processed and analyzed the data. CG obtained access to the database and completed the data extraction. LZ and YZ revised the manuscript and modified the figures and tables. QP designed the study and supervised the study. All authors contributed to the article and approved the submitted version.
