# Supplementary material for: Association between albumin infusion and septic patients with coronary heart disease: A retrospective study based on medical information mart for intensive care III database
Source: Front Cardiovasc Med. 2022 Oct 19;9:982969. doi: 10.3389/fcvm.2022.982969 (PMC9626508; doi:10.3389/fcvm.2022.982969)

Supplementary Table 1. Comparisons of characteristics for patients with albumin infusion or not including mean blood pressure (mmHg), heart rate (r/min), respiratory (r/min), SOFA score, lactate (g/L) before death or discharge.

Supplementary Table 2. Comparisons of characteristics for patients with albumin infusion or not including source of sepsis n (%), albumin infusion dose (g), the first 24 hours fluid infusion (ml), affected organ. Source of sepsis in the type others including tissue infection, positive in blood culture but no details about the site of infection, intracranial infection and the mixed infection.

Supplementary Figure 1 Flow chat of patient inclusion

Supplementary Table 1

Characteristics	Albumin infusion		*P* value
	Yes	No	
Temperature (℃)	36.29(0.74)	36.38 (0.76)	0.110
Mean blood pressure (mmHg)	71.71 (8.36)	72.72 (9.42)	0.138
Heart rate (r/min)	84.94 (13.67)	85.53 (15.19)	0.592
Respiratory (r/min)	18.68 (3.72)	19.18 (4.25)	0.096
SOFA score	5.03 (3.63)	4.83 (3.76)	0.468
Lactate (g/L)	1.87 (1.74)	2.09 (1.78)	0.104

Supplementary Table 2

Characteristics	Albumin infusion		*P* value
	Yes	No	
Source of sepsis n (%), SD			0.001
Respiratory	94 (26.9)	102 (29.2)	
Urinary track	118 (33.7)	98 (28.1)	
Abdominal	58 (16.6)	96 (27.5)	
Others	80 (22.9)	51 (14.6)	
Albumin dose (g)	25.19 (15.38)	0	＜0.001
24 hours fluid infusion (ml)	4674.08 (4214.49)	3689.70 (3235.61)	＜0.001
Organ dysfunction n (%)			0.365
Lung	187 (53.4)	194 (55.4)	
kidney	106 (30.3)	116 (36.1)	
Other organs	55 (15.7)	40 (11.4)	

Supplementary Figure 1

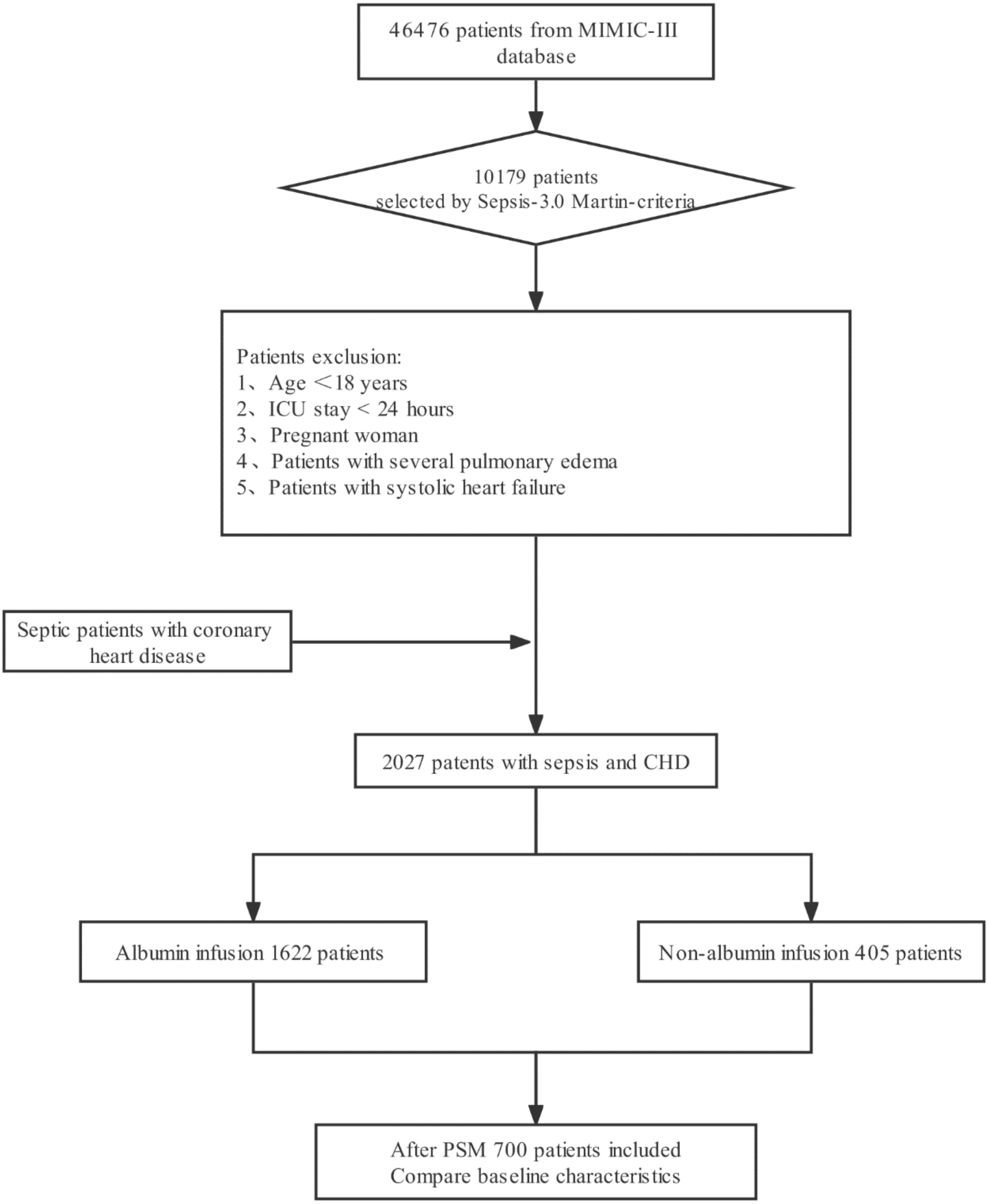

Supplement: Supplementary file 1 [file Data_Sheet_1.pdf]
